# When Missing Data Speak: An Adherence-Driven Sparsity Framework for Patient-Generated Telemedicine Data

**DOI:** 10.2196/92710

**Published:** 2026-11-05

**Authors:** Gábor Kőrösi, Beatrix Rafael, István Kósa

**Affiliations:** 1 Department of Artificial Intelligence, Institute of Informatics, University of Szeged, Árpád tér 2., Szeged, Csongrád, 6720, Hungary, 36 62546396, 36 62546396; 2 Department of Preventive Medicine, Albert Szent-Györgyi Medical School, University of Szeged, Szeged, Csongrád, Hungary

**Keywords:** telemedicine, mobile health, patient-generated health data, patient engagement, missing data, data sparsity, longitudinal studies, real-world data

## Abstract

Telemedicine and mobile health technologies increasingly rely on patient-generated health data collected outside traditional clinical settings. These data promise continuous monitoring, personalization, and early intervention, but in practice, they are often sparse, irregular, and incomplete because data availability depends on sustained patient participation. In this viewpoint, we argue that missingness in patient-generated telemedicine data should not be treated solely as a technical data quality problem. In many contexts, it may reflect patient adherence behavior and, therefore, carry behavioral or clinical meaning. We introduce the adherence-driven sparsity framework, an interpretive and reporting framework for reasoning about missingness in these data. The framework is grounded in 4 principles: patient adherence behavior is a central determinant of data availability, missingness patterns may carry clinically meaningful information, adherence heterogeneity creates differential representation in observed data, and analytical methods should be aligned with actual data characteristics rather than assumed data ideals. The adherence-driven sparsity framework does not propose a new algorithm or validated decision rule. Instead, it provides a structured workflow for preanalysis reasoning: identifying the data generation context; distinguishing adherence-driven from system-driven gaps; characterizing missingness across 6 reporting dimensions; assessing the plausibility of missing completely at random, missing at random, and missing not at random mechanisms; and aligning analytical choices with sparsity, irregularity, heterogeneity, and available evidence. The framework is intended for telemedicine researchers, clinical data scientists, and biostatisticians working with mobile health data. This viewpoint also cautions against the uncritical transfer of machine learning methods from dense, regularly sampled domains to patient-generated telemedicine data. In this setting, methodological sophistication alone is insufficient; researchers must first understand how the data were generated, why observations are missing, and whether missingness itself may be informative. Recognizing adherence-driven sparsity can support more transparent reporting, more defensible analytical decisions, and more clinically meaningful interpretation of patient-generated data.

## Introduction

The past decade has witnessed a fundamental shift in chronic disease management. Digital health technologies—smartphones, connected medical devices, and telemedicine platforms—have enabled health monitoring outside traditional clinical settings [1,2]. Meta-analyses demonstrate meaningful clinical improvements across chronic conditions, including diabetes [3], heart failure [4], and self-management across multiple disease states [5,6]. However, beneath these aggregate successes lies a persistent challenge: patient adherence to monitoring protocols declines systematically over time, and this decline has direct consequences for the data on which clinical insights depend [7,8].

This adherence challenge creates a distinctive data problem. Unlike electronic health records generated through standardized clinical workflows, patient-generated health data depend on patients actively and consistently interacting with monitoring technologies. When engagement declines—as is commonly observed in many digital health deployments [9-11]—datasets become dominated by irregular sampling, substantial temporal gaps, and highly variable completeness across individuals [12,13].

In this paper, we use the term “patient-generated telemedicine data” to refer to health data whose availability depends on sustained patient participation. This includes self-reported symptoms, app-based logs, home device measurements (eg, blood glucose and blood pressure), and wearable data where patient behavior materially affects data availability—for example, by deciding whether to wear, charge, or synchronize a device. We distinguish this scope from passively collected continuous data streams (eg, always-on environmental sensors) and clinician-generated electronic health records. The defining characteristic is that patient behavior directly determines whether, when, and how observations are recorded; our scope is unified by this patient-as-data-source characteristic rather than by any single technology or clinical domain.

The statistical missing data literature has long recognized that missingness mechanisms matter. The foundational taxonomy by Rubin [14] and its subsequent elaboration [15] distinguish data missing completely at random (MCAR), missing at random (MAR), and missing not at random (MNAR). We do not claim that existing methods treat missingness as simple noise; however, in applied telemedicine research, the practical reality often diverges from this methodological sophistication. Missing observations are frequently handled through listwise deletion, imputation under implicit MAR assumptions, or complete-case analysis—often without empirical assessment of whether those assumptions hold for patient-generated data. Sensitivity analyses for MNAR remain rare in published mobile health (mHealth) trials [11]. This operational gap between what the missing data literature recommends and what telemedicine practice typically implements is the starting point for our argument.

This gap is particularly consequential for patient-generated data because the primary driver of data incompleteness—patient adherence behavior—is itself clinically and psychologically meaningful, reflecting dynamic self-regulation rather than purely technical behavior. Patients’ illness representations shape whether they persist with monitoring tasks [16]; perceived competence and self-efficacy influence sustained participation [17,18]; and when the workload of monitoring exceeds patients’ capacity, disengagement becomes a predictable response that may itself reflect clinically meaningful change [19]. Accordingly, when a patient stops logging symptoms, skips scheduled measurements, or gradually disengages from a monitoring protocol, this behavioral pattern may carry information about their health status, motivation, or life circumstances [20,21]—information that standard analytical approaches may discard or obscure.

Importantly, missingness is an ambiguous signal: declining engagement may reflect deterioration (worsening symptoms and increased burden) or improvement (symptoms resolved and monitoring is no longer perceived as necessary). The claim is not that missing data have a single predictable meaning but that they have potential meaning warranting investigation rather than automatic correction.

In this viewpoint, we argue that adherence-driven missingness in patient-generated telemedicine data should be explicitly recognized, systematically characterized, and—where evidence supports it—interpreted as a behavioral signal rather than treated solely as a data quality problem. We aim to (1) articulate why adherence-driven sparsity requires specific consideration in these data beyond generic missing data handling, (2) offer an interpretive and reporting framework for assessing adherence-driven data properties systematically, and (3) provide practical guidance for aligning analytical reasoning with actual data characteristics.

This paper is addressed to telemedicine researchers, biostatisticians working with mHealth data, clinical data scientists, and developers of remote patient monitoring systems.

The remainder of this paper develops this argument in four steps: (1) establishing why patient-generated telemedicine data have distinctive missingness properties and identifying a practice gap in current analytical approaches; (2) formulating 4 principles that constitute the conceptual foundation of the framework; (3) translating these principles into a practical workflow, reporting structure, and illustrative example; and (4) discussing implications, the relationship to existing work, and limitations.

We emphasize that the contribution of this work is not the claim that missingness can be informative—this is well established [14,15] and has recently been discussed in relation to structured missingness in biomedical data [22]. Rather, the contribution lies in integrating missing data reasoning with patient adherence behavior in the specific context of patient-generated telemedicine data (see the Relationship to Existing Work section for a detailed discussion of how the adherence-driven sparsity framework [ADSF] relates to existing work). To our knowledge, no existing framework explicitly centers patient adherence behavior as the organizing principle for interpreting, reporting, and reasoning about missingness in this context. The ADSF, therefore, offers a structured vocabulary for this task—not as a validated decision algorithm but as a conceptual and practical organizing tool for a pervasive challenge in telemedicine research.

## Why Patient-Generated Telemedicine Data Are Different

### From Protocol-Driven to Patient-Driven Data Generation

Traditional clinical data—laboratory values, imaging results, and vital signs recorded during visits—are generated through standardized protocols controlled by health care providers. Measurement timing follows clinical indication, professional judgment, or research schedules, and continuous professional oversight typically limits the influence of patient behavior on data availability.

Patient-generated telemedicine data invert this relationship. The patient controls when, whether, and how data are recorded [21,23]. Device use patterns, self-report compliance, and engagement with monitoring platforms all depend on patient decisions embedded in daily life contexts. This shift introduces behavioral factors as primary determinants of data availability—and, consequently, as potential drivers of missingness mechanisms.

It is important to distinguish 2 sources of data incompleteness. Adherence-driven missingness arises from patient behavior: decisions not to measure, forgetting, disengagement, or intentional avoidance. System-driven missingness arises from technical factors: device malfunction, battery depletion, connectivity failures, or sensor drift. While both produce gaps in the observed record, their implications differ: adherence-driven missingness more directly invites behavioral or clinical interpretation, whereas system-driven missingness first requires technical quality control. In practice, the 2 often co-occur and can be difficult to disentangle—a patient who removes a wearable device exhibits both technical nonwear and behavioral choice.

### Engagement Decline and Its Clinical Correlates

Research consistently documents declining engagement with digital health interventions over time [9,10]. Large-scale analyses involving over 100,000 participants confirm that early engagement patterns strongly predict long-term retention [9], and this decline follows trajectories influenced by patient characteristics, intervention design, and contextual factors [24-26]. Critically, adherence patterns are not merely predictors of data availability—they are associated with health outcomes across chronic pain [27], rheumatoid arthritis [28], smoking cessation [29], mental health [30], and cardiac rehabilitation [31]. App engagement also moderates intervention effectiveness [32].

This evidence points to a structural feature of patient-generated telemedicine data: the data we observe are systematically filtered through an adherence process that may itself carry clinical meaning. Patients who contribute the most data are often those who are most engaged; those whose data are most sparse may be the patients about whom we most need information.

### The Practice Gap

A substantial strand of applied digital health and wearable-sensing research approaches missing observations primarily as a reconstruction or preprocessing problem—asking how accurately missing values can be estimated, replaced, or made usable for downstream modeling [33,34]. We cite such studies not as examples of poor methodology but as illustrations of a legitimate and influential analytical orientation: missingness is treated primarily as a target for reconstruction or preprocessing. This concern is not merely theoretical. Goldberg et al [11] found that differential attrition was common in mHealth randomized trials, yet sensitivity analyses for data MNAR were not reported in the reviewed studies. However, reconstruction is not the same as interpretation. Methods designed to estimate missing values ask what the missing measurement might have been, not why it is missing. A missing entry may reflect forgetfulness, technical failure, symptom worsening, treatment burden, or deliberate disengagement—causes with different clinical and analytical implications even when they produce the same empty cell. If missingness is adherence driven, imputing values without first characterizing the missingness pattern may erase information about patient behavior, differential engagement, or clinical vulnerability. The ADSF complements reconstruction-oriented approaches by shifting the first analytical question from “How should we fill the gaps?” to “What generated these gaps, and should they be interpreted, modeled, reported, or only then reconstructed?”

## Adherence-Driven Sparsity: 4 Principles

### Overview

The data characteristics and practice gap described in the previous section manifest in 4 interconnected challenges for telemedicine data science. We formulate these as principles that, taken together, constitute the conceptual foundation of the ADSF.

Each principle is grounded in established findings: principle 1 draws on documented engagement decline patterns in mHealth [9,10] and the resulting data irregularity [23,35]; principle 2 builds on the foundational missingness taxonomy [14,15] and evidence of differential attrition in mHealth trials [11]; principle 3 extends well-documented population heterogeneity in treatment response [36,37] to its interaction with adherence-driven data availability; and principle 4 responds to concerns about model-data mismatch in health care AI [38,39].

### Adherence as Root Cause: Sparsity and Irregular Temporal Structure

Principle 1: Patient adherence behavior is a central determinant of data availability, timing, and completeness in many forms of patient-generated telemedicine data

Patient-generated data exhibit sparsity across multiple dimensions [23,35]: irregular sampling intervals, missing scheduled measurements, multi-resolution heterogeneity across data streams, and fragmented trajectories with variable-length gaps. These are not incidental data quality issues; they are direct consequences of placing data generation in patients’ hands.

Sampling irregularity degrades the performance of many standard analytical approaches [36]. While several architectures have been designed to handle missing values and irregular sampling directly—including the gated recurrent unit with decay (GRU-D) model [37], convolutional approaches for incomplete data [38], and attention-based methods [39,40]—the challenge in patient-generated telemedicine data is that missingness rates often far exceed those in the datasets on which these architectures were developed, and the missingness process itself may be informative rather than ignorable.

### Missingness as Information: Mechanisms, Patterns, and Interpretation

Principle 2: Patterns of missing data—when, how frequently, and which variables—may carry clinically meaningful information that warrants investigation and transparent reporting rather than erasure through imputation

#### Missingness Mechanisms

The foundational missing data taxonomy [14,15] distinguishes 3 mechanisms. Data are MCAR when the probability of missingness is independent of both observed and unobserved values—for example, a blood glucose meter whose battery dies at unpredictable intervals. Data are MAR when missingness depends on observed variables but not on the unobserved values themselves—for example, when patients with higher baseline symptom severity (an observed quantity) show lower completion rates, but missingness does not additionally depend on their current unrecorded state. Data are MNAR when missingness depends on the unobserved values—for example, when a patient stops logging mood precisely because their current mood has worsened beyond what is captured in the observed record.

Many commonly used likelihood-based and multiple imputation approaches rely on MAR assumptions, whereas complete-case analyses are generally defensible only under stronger assumptions. These assumptions may frequently be violated in patient-generated data. Systematic reviews of mHealth trials document differential attrition, with dropout rates approximately twice as high in active intervention arms vs passive controls [11]. Joint modeling studies quantify engagement-dropout relationships: each additional day of app use is associated with a 23% decreased dropout risk [21]. Joint models with multilevel decomposition distinguish within-person and between-person missingness mechanisms that can operate simultaneously, including nonignorable (MNAR) processes [41]. However, sensitivity analyses for MNAR scenarios remain rare in published mHealth trials [11].

A critical nuance is that the boundary between MAR and MNAR is not always sharp in practice, and many adherence-related missingness patterns may be consistent with MAR when sufficient baseline and contextual covariates are available. The claim is not that all adherence-driven missingness is MNAR but that the plausibility of MNAR should be explicitly assessed rather than dismissed by default.

#### Missingness Patterns and Structured Missingness

The temporal characterizations relevant to patient-generated data—random gaps, systematic decay, episodic bursts, and context-clustered silence—complement standard structural classifications such as monotone, nonmonotone, and file-matching patterns [42]. Monotone patterns are particularly common in patient-generated data as dropout creates irreversible data loss, but many patients exhibit nonmonotone patterns with intermittent re-engagement. Recent work on structured missingness [22] recognizes that such patterns often carry systematic structure that can be leveraged rather than merely corrected. We build on this perspective by focusing on a specific telemedicine context in which the structure of missingness is generated by patient adherence behavior and may therefore be clinically interpretable. Importantly, missingness remains an ambiguous signal—disengagement from educational self-tracking may reflect learning, whereas disengagement from therapeutic monitoring may have different implications—and interpretation requires triangulation across baseline characteristics, concurrent observations, and temporal context.

### Heterogeneity Amplification

Principle 3: Adherence patterns vary systematically across patients, amplifying population heterogeneity in observed data and creating differential representation

Patients differ substantially in adherence patterns, disease trajectories, and treatment responses. This heterogeneity interacts with adherence-driven sparsity to create compounding analytical challenges. Population-average effects frequently fail to predict individual responses [43,44], and person-specific analyses reveal that relationships observed at the population level may not hold, or may even reverse, at the individual level [44].

Heterogeneity amplifies the sparsity problem in 2 ways. First, naive pooling of sparse observations may obscure person-specific relationships, and individual-level interpretation may require methods that preserve within-person temporal structure. Second, different patients may exhibit different missingness mechanisms: some disengage when symptomatic, others disengage when doing well. This heterogeneous missingness violates uniform assumptions underlying standard imputation approaches [11,45] and means that a single missingness-handling strategy may be inappropriate for the entire sample.

The practical consequence is differential representation: high-adherence patients contribute dense trajectories that dominate analyses, whereas low-adherence patients contribute sparse fragments that may be excluded or downweighted. Models built on such data risk reflecting only the experience of engaged patients and failing to generalize to the broader patient population.

### Model-Data Alignment

Principle 4: Analytical methods should be selected based on actual adherence-driven data characteristics, not assumed data ideals

Contemporary health care AI has pursued increasingly complex architectures—deep learning, transformers, and multimodal fusion—that typically require large, high-quality datasets [46]. Patient-generated telemedicine data may instead involve small samples, high sparsity, heterogeneous populations, and potentially informative missingness [11,13,47].

This mismatch has practical consequences. Models may achieve high apparent performance by exploiting informative missingness patterns rather than true clinical signals, undermining transportability when missingness patterns change across settings [48]. The interpretability required for clinical adoption may conflict with the opacity of sophisticated architectures [49]. In many sparse or small-sample clinical settings, simpler interpretable models may be competitive with more complex deep learning approaches [47,50].

The alignment calls for honest assessment of actual data characteristics. A central concern is the uncritical transfer of methods from domains with dense, regularly sampled data (eg, imaging and financial time series) into patient-generated telemedicine research, where observations are sparse, irregular, and shaped by adherence—methodological sophistication alone is insufficient without alignment with the data generation process.

## From Principles to Practice

The 4 principles outlined above provide conceptual grounding. This section translates them into a practical interpretive workflow: a structured process for characterizing, reporting, and reasoning about adherence-driven sparsity before selecting analytical methods.

### An Interpretive Workflow

The ADSF workflow proceeds through 3 stages. Figure 1 provides an overview.

**Figure 1.**
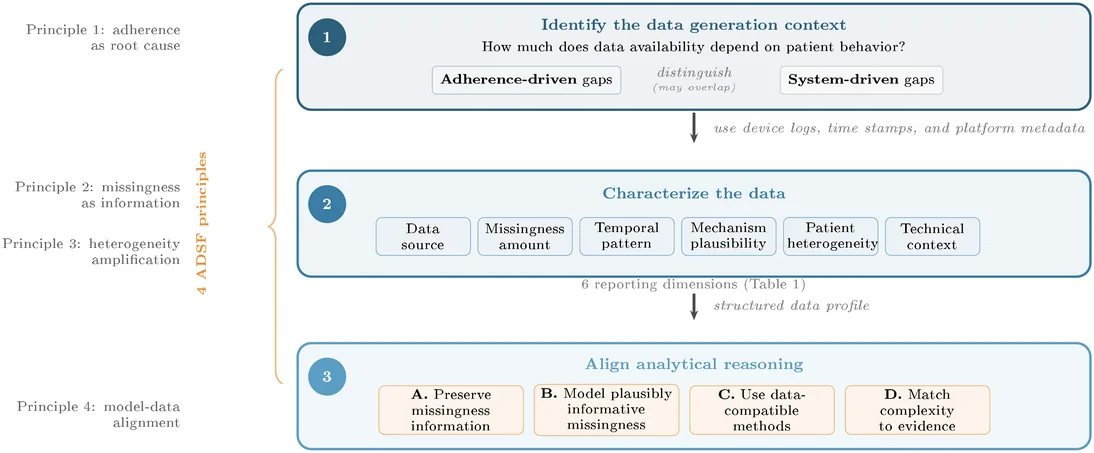
The adherence-driven sparsity framework (ADSF): an interpretive workflow proceeding through 3 stages—identifying the data generation context, characterizing the data across 6 reporting dimensions, and aligning analytical reasoning with the resulting data profile. The ADSF is not intended as a validated decision algorithm.

Stage 1 (identifying the data generation context) determines the extent to which data availability depends on patient behavior and distinguishes adherence-driven gaps (the patient did not log in, removed the device, or disengaged) from system-driven gaps (server downtime, battery failure, or connectivity loss) using device logs, time stamps, or platform metadata where available.

Stage 2 (characterize the data) systematically assesses data properties using the reporting dimensions described in the Data Characterization: A Reporting Framework section, producing a structured profile that makes adherence-driven sparsity visible and reportable.

Stage 3 (align analytical reasoning) uses the data profile to guide methodological choices following the principles in the Four Methodological Principles section, ensuring that analytical decisions are defensible given the characterized data properties.

### Data Characterization: A Reporting Framework

#### Overview

Table 1 shows 6 dimensions for systematically characterizing adherence-driven data properties intended for structured reporting rather than as decision thresholds.

**Table 1.** Reporting dimensions for adherence-driven sparsity in patient-generated telemedicine data.

Dimension	What to report	Why it matters	Possible analytical response
Data source	Active logging, home device, wearable, and questionnaire	Determines the role of patient behavior in data generation	Distinguish system-driven from adherence-driven gaps
Missingness amount	Per-patient and per-variable completeness rates	Reveals the sparsity burden and its distribution	Assess whether complete-case or imputation-based approaches risk bias
Temporal pattern	Random gaps, systematic decay, episodic bursts, and dropout	Suggests whether missingness follows a behavioral process	Include gap features or dropout timing in analysis; consider monotone vs nonmonotone patterns
Mechanism plausibility	Evidence relevant to MCAR^a^, MAR^b^, and MNAR^c^ plausibility	Shapes the validity of analytical assumptions	Conduct sensitivity analyses under alternative mechanism assumptions
Patient heterogeneity	Between-patient variation in completeness and trajectories	Affects whether pooling is appropriate	Consider subgroup, hierarchical, or person-specific approaches
Technical context	Battery logs, connectivity records, and device failure rates	Separates technical artifacts from behavioral signals	Apply technical quality control before interpreting missingness behaviorally

a MCAR: missing completely at random.

b MAR: missing at random.

c MNAR: missing not at random.

Researchers should report these dimensions explicitly in their methods sections. The existing literature offers various proportion-based guidelines—for example, deletion may be acceptable below approximately 5% missingness [51], while simple imputation may perform adequately up to 15-20% [52], with model-based approaches recommended for higher rates. However, in patient-generated telemedicine data, where missingness can be substantial and the mechanism uncertain, proportion alone is an insufficient guide. We emphasize mechanism plausibility, temporal pattern, and data generation context over fixed thresholds.

#### Assessing Mechanism Plausibility

As MNAR cannot be directly tested from observed data alone, researchers must rely on indirect evidence. Useful indicators include whether patients with worse baseline values show higher subsequent missingness, which is consistent with MAR if baseline values are observed and suggestive of MNAR only if the association persists after conditioning on relevant observed covariates; whether gaps cluster before adverse clinical events, which may suggest MNAR when the current state is unobserved; whether missingness patterns correlate with external data sources such as emergency visits or prescription records; and whether formal tests such as the Little MCAR test provide evidence against MCAR assumptions. Even with proxy evidence, MNAR cannot be confirmed with certainty, and MAR and MNAR generally cannot be definitively distinguished from observed data alone. Sensitivity analysis is therefore essential; pattern mixture models and tipping point analyses provide established frameworks for such assessment [11,15].

### Four Methodological Principles

The following methodological principles operationalize the 4 conceptual principles described in the Adherence-Driven Sparsity: 4 Principles section: principle A follows from recognizing missingness as information (principle 2); principle B follows from mechanism plausibility (principle 2); principle C follows from sparsity, irregularity, and heterogeneity (principles 1 and 3); and principle D follows from model-data alignment (principle 4). These principles illustrate compatible analytical reasoning rather than prescribing specific algorithms.

#### Principle A: Preserve Missingness Information

Before imputing or discarding incomplete observations, consider whether missingness patterns themselves carry analytical value. Gap duration, time since last observation, per-patient completion trajectories, and modality-specific silence patterns can be included as features or covariates. Preserving this information allows downstream analyses to leverage adherence-driven signals rather than erasing them.

#### Principle B: Model Missingness When It Is Plausibly Informative

When there is evidence suggesting that missingness may be related to the outcome of interest (ie, MNAR is plausible), consider approaches that explicitly model the missingness process—for example, joint longitudinal survival models, pattern mixture models, or selection models [15]. At minimum, sensitivity analyses comparing results under MAR vs MNAR assumptions should be reported.

#### Principle C: Use Methods Compatible With Irregular and Sparse Data

Select approaches that can accommodate the temporal structure of patient-generated data without requiring aggressive preprocessing. Examples of compatible approaches include mixed-effects models for unbalanced designs, Gaussian processes for irregular sampling with uncertainty quantification, continuous time models (eg, neural ordinary differential equations) that treat irregular intervals as a feature, and set function approaches for extreme sparsity. Simple feature-based approaches may be effective and transparent when sample sizes are limited.

#### Principle D: Match Model Complexity to Available Evidence

Resist the temptation to apply the most sophisticated available architecture without assessing whether the data can support it. Deep temporal models, transformers, and transfer learning approaches often require substantial data, careful validation, and architectures explicitly suited to irregular or sparse observations. When sample sizes are small or heterogeneity is high, simpler interpretable models may be both more robust and more informative.

### Illustrative Example: Smartphone-Based Depression Monitoring

The following is a hypothetical illustrative example with plausible but simplified values intended to demonstrate ADSF reasoning rather than report empirical findings. Consider a 12-week smartphone-based depression monitoring study with 200 participants completing daily mood logs, with baseline Patient Health Questionnaire–9 (PHQ-9) scores available.

Data characterization is as follows. Data source is active daily mood logging via smartphone app, indicating high dependence on patient behavior. Missingness amount is 58% of expected entries, with per-patient completeness ranging from 4% to 100% (mean 42%). Temporal pattern is declining completion over weeks 6 to 12 in most participants, with a subset showing episodic re-engagement (systematic decay with nonmonotone elements). In terms of mechanism plausibility, higher baseline PHQ-9 scores are associated with higher dropout (odds ratio 1.8; *P*<.01). This pattern is compatible with MAR because baseline PHQ-9 is an observed variable. However, missing entries also cluster before clinical deterioration events (hospitalizations and crisis contacts), raising concerns that missingness may additionally depend on the patient’s evolving but unobserved symptom state—making MNAR plausible. In this scenario, clinical deterioration and adherence are linked rather than independent: worsening symptoms may directly reduce the patient’s capacity or motivation to engage with monitoring, making the resulting missingness adherence driven rather than random. The systematic clustering of gaps before clinical events is inconsistent with MCAR, which requires that missingness probability is independent of both observed and unobserved values. This does not prove MNAR but justifies sensitivity analyses under MNAR assumptions. Device logs show that 12% of gaps coincide with server downtime or app crashes (system driven). In terms of patient heterogeneity and technical context, the intraclass correlation coefficient for mood trajectories is 0.62 (substantial between-person variability); server logs confirm that 12% of gaps are technical.

Analytical reasoning (following principles A-D) involves including gap duration, completion slope, and days since the last entry as features (principle A); fitting a joint longitudinal survival model with sensitivity analyses under MAR vs MNAR (principle B); using the Gaussian process or mixed-effects models as primary analysis for irregular observations (principle C); and, with N=200, prioritizing interpretable probabilistic approaches over complex deep learning architectures (principle D).

This example illustrates the intended role of the ADSF: not to select a method automatically but to make visible the assumptions and behavioral interpretations that should precede method selection.

To illustrate the difference that ADSF-guided reasoning can make, consider an alternative analysis of the same data that follows standard practice without systematic missingness assessment. A researcher might apply multiple imputation under a default MAR assumption without separating the 12% system-driven gaps from adherence-driven gaps, without assessing whether the baseline severity-dropout association fully accounts for the missingness pattern, and without preserving gap duration or completion trajectories as analytical features. Under this approach, the association between symptom worsening and predeterioration disengagement would be treated as missing information to be reconstructed rather than as a potential behavioral signal to be investigated. The ADSF does not guarantee different conclusions, but it ensures that such decisions are made explicitly rather than by default.

## Implications and Limitations

### Implications for Research Practice

The ADSF has practical implications across the research life cycle.

#### Study Design

Engagement patterns should be considered potential process outcomes, not merely determinants of data quality, and studies should prespecify how missingness will be characterized and reported. Design decisions should balance data density against sustainable engagement, recognizing that highly complete data from intensively supported settings may not generalize to real-world deployments where patients manage monitoring independently.

#### Data Analysis

We recommend the systematic workflow described in the From Principles to Practice section: characterize data properties before selecting methods, assess mechanism plausibility rather than assuming MAR by default, evaluate whether missingness patterns should be preserved as features, select methods aligned with actual data characteristics, and report missingness-related decisions transparently.

#### Clinical Deployment

If declining engagement is associated with adverse outcomes, monitoring engagement trajectories may enable clinical response before patients disengage entirely [21,27,31]. The ADSF perspective suggests that nonresponse may warrant clinical attention—not because all silence indicates crisis but because silence should be characterized and interpreted in context rather than automatically corrected. This reframing has implications for how monitoring systems are designed and how alerts are triggered.

### Relationship to Existing Work

The ADSF draws on and integrates several established lines of research. The missing data literature [14,15] provides the foundational taxonomy of missingness mechanisms. The concept of structured missingness [22] recognizes that missingness patterns in biomedical data can carry systematic, leverageable structure. The mHealth engagement literature [12,13] documents the prevalence and clinical correlates of adherence-driven data loss. Finally, the behavioral science perspective [16,18] explains why engagement is not merely a technical behavior but a psychologically meaningful process.

Among these lines of research, Scherer et al [21] demonstrated that engagement and dropout can be jointly modeled using shared random effects, providing important empirical evidence that missingness and engagement are statistically linked in mHealth data. The ADSF builds on this insight but operates at a different level: rather than proposing a specific statistical model, it provides a preanalysis workflow for determining which analytical approach—including but not limited to joint modeling—is appropriate given the characterized data properties.

The contribution of the ADSF is not to any of these individual areas but to their integration in a specific applied context. By connecting behavioral understanding of why patient-generated data are incomplete with methodological reasoning about how to analyze them, the framework addresses a gap that exists between disciplines rather than within them. The relevant knowledge exists across these disciplines; what has been lacking is a structured workflow that integrates behavioral understanding of why data are incomplete with methodological reasoning about how to analyze them in the specific context of patient-generated telemedicine data. The ADSF provides this integration through a shared vocabulary and a systematic preanalysis process.

### Limitations

Several limitations warrant acknowledgment. First, the ADSF is a conceptual and interpretive framework that has not been empirically validated through comparative studies. Future research should evaluate whether ADSF-guided reasoning leads to more defensible conclusions than standard approaches across diverse telemedicine datasets.

Empirical demonstration of the ADSF using real-world telemedicine datasets is an important direction for future research. Such work could compare analytical conclusions reached with and without systematic adherence-driven sparsity assessment, providing evidence for the framework’s practical value.

Second, the framework does not provide software implementations, and effective application requires both statistical expertise and clinical domain knowledge. Assessing missingness mechanisms, implementing sensitivity analyses, and interpreting ambiguous patterns are nontrivial tasks that may limit adoption in resource-constrained settings.

Third, not all missingness in patient-generated data is behaviorally meaningful. The framework may encourage overinterpretation of gaps that are random or technical; the reporting dimensions in Table 1—particularly the technical context dimension—guard against this, but the risk remains. Relatedly, the framework’s applicability may vary across clinical domains, measurement modalities, and patient populations, and domain-specific adaptations are needed.

Fourth, interpreting missingness as a potential clinical signal has ethical and operational implications. False alarms based on engagement decline may increase clinician workload or patient burden, whereas missed signals may create safety risks. Any clinical use of adherence-based alerts requires prospective validation, appropriate safeguards, and careful consideration of patient autonomy and privacy.

## Conclusions

Patient-generated telemedicine data present analytical challenges that differ from those in clinician-controlled settings. Data sparsity is not incidental: it reflects the fundamental nature of patient-driven data collection. Missingness may carry information about patient behavior, engagement, and health status, whereas heterogeneity in adherence patterns can create differential representation in observed data. Analytical methods must therefore be selected with these data generation realities in mind rather than applied as if the data were dense, regular, and technically generated.

The central argument of this viewpoint is not simply that missingness can be informative; this is well established in the statistical literature. Rather, we argue that patient-generated telemedicine data have their own data generation logic rooted in patient adherence behavior and that this logic should be examined before analytical methods are applied. In this context, missingness is not only a statistical mechanism to be modeled but also the possible product of a behavioral process that may be clinically interpretable. Recognizing this requires researchers to characterize the origins of data incompleteness by distinguishing behavioral from technical gaps, assessing mechanism plausibility, and considering patient heterogeneity before proceeding to method selection.

The ADSF offers an interpretive and reporting structure for this task. It encourages researchers to characterize before analyzing, assess mechanism plausibility before imputing, preserve missingness information where it may be informative, and match analytical complexity to the evidence at hand. The framework does not solve the challenges of telemedicine data science: sparsity, potential MNAR mechanisms, heterogeneity, and small samples remain genuine obstacles. However, it provides a shared vocabulary for recognizing these challenges, reporting them transparently, and reasoning about them systematically.

As telemedicine expands into routine care, the gap between controlled research settings and real-world deployment will become increasingly important. Methods developed on curated, complete datasets may struggle when confronted with sparse, irregular patient-generated data. In this setting, missingness should not automatically be removed through correction. In many contexts, it should first be characterized, reported, and interpreted.

## Abbreviations

ADSF: adherence-driven sparsity framework
GRU-D: gated recurrent unit with decay
MAR: missing at random
MCAR: missing completely at random
mHealth: mobile health
MNAR: missing not at random
PHQ-9: Patient Health Questionnaire–9
